# Segmental ureterectomy for high-risk ureteral carcinoma: a preliminary report

**DOI:** 10.1186/s12894-023-01265-y

**Published:** 2023-06-05

**Authors:** Wei Wei, Junfeng Liu, Lingdian Wang, Xiaoyu Duan, Degang Ding

**Affiliations:** Department of Urology, Henan Provincial People’s Hospital, Zhengzhou University People’s Hospital, Zhengzhou, China

**Keywords:** High-risk, Kidney sparing surgery, Segmental ureterectomy, Ureteral carcinoma

## Abstract

**Background:**

EAU guidelines strongly recommend kidney sparing surgery (KSS) as the primary treatment option for the low-risk UTUC patients. While there are few reports involving the KSS treated for the high-risk counterparts, especially the ureteral resection.

**Objective:**

To evaluate the effectiveness and safety of the segmental ureterectomy (SU) for the patients with high-risk ureteral carcinoma.

**Materials and methods:**

We included 20 patients from May 2017 to December 2021 who underwent segmental ureterectomy (SU) in Henan Provincial People’s Hospital. The overall survival (OS) and progression free survival (PFS) were evaluated. Besides, the ECOG scores and postoperative complications were also included.

**Results:**

As of December 2022, the mean OS was 62.1months (95%CI:55.6-68.6months) and the mean PFS was 45.0months (95%CI:35.9-54.1months). The median OS and median PFS were not reached. The 3-year OS rate was 70% and the 3-year PFS rate was 50%. The percentage of Clavien I and II complications was 15%.

**Conclusion:**

For the selected patients with high-risk ureteral carcinoma, the efficacy and safety of segmental ureterectomy were satisfactory. But we still need to conduct prospective or randomized study to validate the value of SU in patients with high-risk ureteral carcinoma.

## Introduction

Urothelial carcinoma (UC) is one of the most common tumors in the whole world, including the bladder cancer (BCa) and upper tract urothelial carcinoma (UTUC). UTUC is a relatively rare type of UC, which accounts for 5–10% of the disease, with an incidence of about 2/100,000 in western countries [1].UTUC can be classified into low-risk and high-risk, for the purpose of making better clinical decisions [2]. Low-risk UTUC must meet all the following conditions: unifocal disease, tumor size < 2 cm, negative for high-grade cytology, low-grade URS biopsy, no invasive aspect on CT; while high-risk UTUC only need to meet any of the following conditions: multifocal disease, tumor size ≥ 2 cm, high-grade cytology, high-grade URS biopsy, local invasion on CT, hydronephrosis, previous radical cystectomy for high-grade bladder cancer and histological subtype. Kidney sparing surgery (KSS) can be considered in low-risk patients, which can achieve the similar oncologic outcomes, and has a lower morbidity rate compared with radical nephroureterectomy (RNU) [3]. In principle, RNU is recommended for high-risk patients, including nephrectomy, ureterectomy, and bladder cuff excision. Nevertheless, some specific patients with contraindications such as solitary kidney, CKD, and multiple comorbidities, are unable to tolerate the radical surgery, which greatly influence the quality of life [4]. At the moment, there are some reports reporting the patients who underwent the KSS, but in these reports some of the people are not the high-risk ones. Some have underwent other surgical procedures instead of ureteral resection. In order to inquire into the efficacy and safety of the segmental ureterectomy (SU), we carry out the research, and we hope that this study can provide some reference for the treatment of this type of the patients.

## Materials and methods

The study was approved by the Ethics Committee of Henan Provincial People’s Hospital. We retrospectively analyzed all patients who underwent KSS procedure in Henan Provincial People’s Hospital from May 2017 to September 2021. All patients with ureteral carcinoma were diagnosed by ureteroscopy, cross-sectional imaging or urine cytology. According to the EAU guidelines of UTUC, we excluded all the low-risk patients and included the high-risk counterparts. ALL of them were followed up for at least 1 year and they did not receive neoadjuvant therapies. To ensure the consistency of the study, we only included patients with segmental ureterectomy, and other surgical methods such as endoscopic ablation were excluded. The procedure of SU is as follows: First, we evaluated the tumor location by imaging or ureteroscopy before surgery, then we performed ureterectomy with or without bladder cuff excision by open or laparoscopic procedure, and we performed ureteroureterostomy or ureteral reimplantation in light of specific conditions. All the surgeries were operated by well-experienced practitioner. Prior to the surgery, patients were well informed about the advantages and disadvantages of the operation. We collected the baseline clinical data from the EMR database. The tumor was categorized according to the 2007 TNM staging system and the 2004 WHO grading system, based on Ruvolo C‘s report [5]. Blood loss was estimated by the surgeon in the OR during the surgery. Operative time was collected according to the surgical records. Postoperative complications were recorded and graded according to the Clavien–Dindo grading system. All patients were followed up every 12 weeks, otherwise they would receive the follow-up calls, we assessed the quality of life (QOL) according to the Eastern Cooperative Oncology Group (ECOG) score.

All *p-*values were two-tailed and *p* < 0.05 was considered statistically significant. The primary endpoint was overall survival (OS), and the secondary endpoints included progression-free survival (PFS), quality of life and postoperative complications. We used the Kaplan-Meier method to analyze the data. All analyses were performed with SPSS25.0 software.

## Results

### Clinical characteristics

From May 2017 to September 2021, we included 20 patients who underwent SU, including 15(75%) males and 5(25%) females. The median age was 69 years (range:20–85 years). All of them showed hydronephrosis on computed tomography (CT) scan. Three patients had a history of BCa, and two patients had ureteral cancer combined with BCa. The median eGFR (calculated by CDK-EPI equation with preoperative creatinine) was 91.74ml/min/1.73m^2^. Table 1 lists all the relevant clinical parameters.

**Table 1:** Demographic and clinical characteristics of patients

Characteristics	SU(n = 20)
Gender, n(%)	
Female	5(25%)
Male	15(75%)
BMI,kg/m^2^	
Median	25.00
Mean ± SD	24.90 ± 0.82
P25, P75	22.50,26.00
Lateral, n(%)	
Right	9(45%)
Left	11(55%)
Comorbidity, n(%)	
None	6(30%)
Hypertension	8(40%)
Diabetes	4(20%)
Coranary heart disease	3(15%)
Chronic kidney disease	3(15%)
Other cancer	1(5%)
Solitary kidney	2(10%)
Preoperative eGFR, ml/min/1.73m^2^	
Median	91.74(12.61-128.03)
Mean ± SD	86.02 ± 6.87
P25, P75	77.94,104.18
Age	
Median	69.00
Mean ± SD	63.5 ± 3.84
P25, P75	60.50,74.00
Tumor size	
＜2cm	4(20%)
≥ 2cm	16(80%)
Smoking history, n(%)	8(40%)
History of BCa,n(%)	3(15%)

Abbreviation:BCa,bladder cancer;BMI,body mass index;GFR,glomerular filtration rate

### OS and PFS

**Fig. 1:**
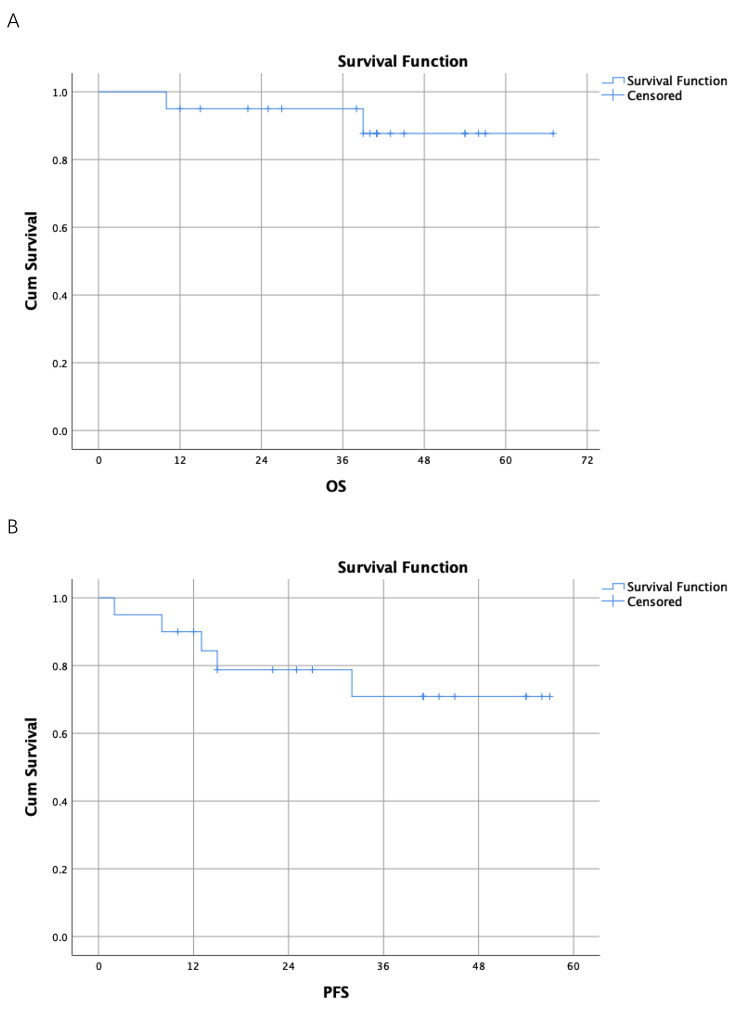
**(A)** Kaplan–Meier curve of OS. **(B)** Kaplan–Meier curve of PFS

We followed up all the patients until the date of December 31, 2022.The mean OS was 62.1months (95%CI:55.6-68.6months) and the mean PFS was 45.0months (95%CI:35.9-54.1months). The median OS and median PFS were not reached. The 3-year OS rate was 70% and the 3-year PFS rate was 50%. During the follow-up, we found that none of patients went through local recurrence and did not underwent RNU. While 5 patients suffered intravesical recurrence, and one of them underwent radical cystectomy. The Kaplan-Meier survival curves for OS and PFS are shown in Fig. 1.

### Operation-related data and QOL

The mean operative time was 199.7 ± 69.9 min.The median blood loss was 35ml.The mean length of hospital stay was 19.7days. Considering the TNM, 12 (60%) patients were categorized as Ta, and 1(5%) patient was staged as T1. The N stage was evaluated by CT, and all the patients had no lymph node metastasis. 2(10%) patients developed Clavien I complications postoperatively and 1(5%) patient developed Clavien II complications postoperatively. The major postoperative complication was fever. Symptoms improved significantly after appropriate treatment. While only 1 patient developed hemorrhagic shock and underwent secondary surgery. Pathological and operation-associated outcomes are shown in Table 2. We evaluated the quality of life with the ECOG score. The QOL is listed in Table 3.

**Table 2:** The pathologic and operative outcomes

T stage	
Ta T1 T2 T3 T4	12(60%) 1(5%) 5(25%) 2(10%) 0
N stage	0
CIS	0
Pathological grade	
Low grade High grade	10(50%) 10(50%)
Estimated blood loss(ml)	
Median Mean ± SD P25, P75	35.00 50.00 ± 10.13 20.00,50.00
Operation time(min)	
Median Mean ± SD P25, P75	173.00 199.70 ± 69.9 149.25,255.00
Complication Grade	
Clavien–Dindo I Clavien–Dindo II Clavien–Dindo III Clavien–Dindo IV Clavien–Dindo V None	2(10%) 1(5%) 1(5%) 0 0 0
Biopsy before operation	
Cytology Ureteroscopy None	1(5%) 12(60%) 7(35%)
Biopsy pathology	
PUNLMP Low grade High grade NO defined NO	5 4 2 2 7
Hospital Stay(Days)	
Median Mean ± SD P25, P75	19.00 19.70 ± 1.56 14.25,23.25.

Abbreviation:CIS,carcinoma in situ;PUNLMP,papillary urothelial neoplasm of low malignant potential

**Table 3:** The QOL of the patients

ECOG score	Preoperative	Postoperative^a^
0 1 2 3 4 5	4(20%) 5(25%) 10(50%) 1(5%) 0 0	4(20%) 3(15%) 11(55%) 1(5%) 1(5%) 0

Abbreviation: ECOG, Eastern Cooperative Oncology Group

a: Collected 6months after surgery

## Discussion

The morbidity of UTUC is relatively rare but aggressive. Compared with bladder cancer,50% of UTUC are invasive. According to tumor location, UTUC could be classified into renal pelvic carcinoma (2/3) and ureteral carcinoma (1/3). Although ureteral carcinoma has similar characteristics to pelvic carcinoma, the patients suffered from ureteral carcinoma have a worse prognosis [6]. Radical nephroureterectomy (RNU) with bladder cuff excision is the gold standard for UTUC, while the procedure has longer operative time, more trauma, higher relative risk, and is physically demanding for surgical patients. Meanwhile, Masaki Momota et al [7] reported that preoperative renal insufficiency is a potential risk factor for poor oncological outcomes in patients with UTUC who underwent RNU. Consequently, some selected patients can choose KSS surgery, including segmental ureterectomy (SU), ureteroscopy (URS), and percutaneous access (PC). The validity and safety of the KSS surgery for low-risk patients have been authenticated, but there are few reports targeting the high-risk ones. We conducted the present study to explore the efficacy of SU in the high-risk group.

The treatment of UTUC was systematic. Surgery was only one important step in the overall treatment protocol, while adjuvant chemotherapy and neoadjuvant chemotherapy were equally important for the patients. The POUT trial [8] corroborated that adjuvant chemotherapy for the UTUC patients after receiving RNU significantly improved disease-free survival (hazard ratio 0.45, 95% CI 0.30–0.68; P = 0.001) at a median follow-up of 30.3months. As for neoadjuvant chemotherapy, there was a study [9] confirming that among all the people who received neoadjuvant chemotherapy, the patients who achieved pathologic complete and partial responses could get improved PFS and OS compared with no responders (≥ ypT2N any; 2-year PFS 100% and 95% vs. 76%, P < 0.001; 2-year OS 100% and 100% vs. 80%, P < 0.001).Nevertheless, owning to the loss of the renal unit, some patients could not tolerance the chemotherapy after receiving the RNU. Xylinas, et al [10] found that the median eGFR decreased by 18% after RNU. The percentage of patients with preoperative eGFR60 ≥ mL/min/1.73 m^2^ ranged from 37 to 16% after RNU (P＜0.001). Correspondingly SU has less impact on kidney function. A meta-analysis [11] showed that the risk of renal function impairment was significantly reduced after SU compared with RNU (mean eGFR difference = 9.32 ml/1.73 m^2^, P = 0.007).

The EAU guidelines [2] recommend KSS as the preferred approach for low-risk patients, with survival similar to that after RNU, and lower mortality rates. For high-risk patients, there have been a few related reports with paradoxical conclusions. Hendriks N et al [12] reported there were no statistical differences in PFS (RNU 96.0%; KSS 86.0%), MFS (RNU 72.0%; KSS 84.0%), CSS (RNU 84.0%; KSS 86.0%), and OS (RNU 76.0%; KSS 76.0%) between high-risk and low-risk groups stratified by EAU guidelines after propensity weight matching. Nevertheless, another report [13] showed those patients with high-grade tumors had a higher relapse rate than those with low-grade tumors. In addition, Collà Ruvolo C [14] found tumor size could linearly predict the rate of muscle-invasive or non-organ-confined RPUC. As a consequence, KSS for high-risk patients should be in a prudent consideration. In our report, the mean OS and PFS were 62.1 and 45 months, respectively. The discrepancies in these reports indicate that the existing evaluation system may be poorly discriminative. Some scholars had proposed a new model [15] for the improvement of decision making for KSS in UTUC in comparison with the current risk stratification systems.

KSS for UTUC include segmental ureterectomy (SU), ureteroscopy (URS), and percutaneous access. Each option has its own advantages and disadvantages. In our center, all cases were selected for SU, based on the following reasons: complete resection of the focus, relatively precise clinical and pathologic staging, and lymphadenectomy if necessary. One study [16] showed that 65.5% of the patients who underwent URS biopsy were in discordance with the final pathologic findings after RNU. Another report [17] confirmed that 85.5% of patients who received SU could achieve 5-year local recurrence-free survival compared with 35.7% of the people who received endoscopic surgery. As for lymphadenectomy, the conclusions of published reports were contradictory, and the treatment effect of lymphadenectomy was controversial [18]. What’s more, many patients in our report could not endure the surgery. Based on the current inconsistent views and the actual conditions of the patients, we chose SU without lymphadenectomy. The key point of SU was the negative surgical margins, which we could identify the tumor location by radiological examination and ureteroscopy before SU, and in a pinch, we could diagnose in the aid of intraoperative frozen section.

The surgical complications induced by KSS is apparently less than those caused by RNU. In our report, the percentage of Clavien I and II complications was 15%. The most common complication was fever and only one patient received reoperation. All these patients were in remission without deterioration after symptomatic treatment. Although surgery site infection and postoperative sepsis are common in non-elective procedures [19] [20], and could increase morbidity, prolong postoperative hospital stay, readmission, and even death. However, the patients in our report did not suffer from these complications, owning to laparoscopic procedure. These were similar to Mason’s report [21], in which 33.4% of the patients had a postoperative complication. The percentage of Clavien I and II complications was 24.5%, including 6.4% of patients receiving reintervention, and 2.5% of life-threatening complications. Meanwhile, they found the length of stay in the medical center was determined by the 7-day stay before removal of the ureter. In our report, the mean length of hospitalization time was 19.7 days, which was the same reason with Mason’s report [21].

The QOL of the patients was minimally affected by SU. Most of the patients had the same ECOG score before and after surgery. Only one patient had an ECOG score of 4,who had a preoperative creatinine of 412µmol/L, and the patient was reluctant to receive RNU, considering the further deterioration of renal function.

There were definitely existing limitations in our study. First, owing to the retrospective nature, the sample size was relatively small. Second, some of the objects had comorbidities, such as solitary kidney or CKD, which were subjected to choose KSS. At the same time, most of the patients underwent ureteroscopic biopsy, in which papillary urothelial neoplasia of low malignant potential (PUNLMP) and low-grade tumor predominated. These cases induced selection bias. However, as previously mentioned, biopsy specimen could not represent the gross specimen in pathology. And our report also indicated the poor discrimination of the existing evaluation system. Furthermore, we chose SU as the only treatment for the patients, which precluded the interference of the other KSS. It was prudent to assume that our report would provide some reference for such patients.

## Conclusion

In summary, we performed SU for the selected patients with high-risk ureteral carcinoma. Although comorbidities were present in some patients, the overall prognosis was satisfactory. It implied that SU was an alternative way for high-risk patients with close scrutiny. Moreover, what we need is to conduct prospective or randomized study to validate the value of SU in patients with high-risk ureteral carcinoma.

## Data Availability

The datasets used and/or analyzed during the current study are available from the corresponding author on reasonable request.

## Abbreviations

BCa: Bladder cancer
UC: Urothelial carcinoma
KSS: Kidney sparing surgery.
SU: Segmental ureterectomy
BMI: Body mass index
GFR: Glomerular filtration rate
ECOG: Eastern Cooperative Oncology Group
CIS: Carcinoma in situ
PUNLMP: Papillary urothelial neoplasm of low malignant potential
